# High Efficacy of Saliva in Detecting SARS-CoV-2 by RT-PCR in Adults and Children

**DOI:** 10.3390/microorganisms9030642

**Published:** 2021-03-19

**Authors:** Michael Huber, Peter Werner Schreiber, Thomas Scheier, Annette Audigé, Roberto Buonomano, Alain Rudiger, Dominique L. Braun, Gerhard Eich, Dagmar I. Keller, Barbara Hasse, Jürg Böni, Christoph Berger, Huldrych F. Günthard, Amapola Manrique, Alexandra Trkola

**Affiliations:** 1 Institute of Medical Virology, University of Zurich, 8057 Zurich, Switzerland; audige.annette@virology.uzh.ch (A.A.); boeni.juerg@virology.uzh.ch (J.B.); huldrych.guenthard@usz.ch (H.F.G.); manrique.amapola@virology.uzh.ch (A.M.); 2 Division of Infectious Diseases and Hospital Epidemiology, University Hospital Zurich, University of Zurich, 8091 Zurich, Switzerland; peterwerner.schreiber@usz.ch (P.W.S.); thomas.scheier@usz.ch (T.S.); Dominique.Braun@usz.ch (D.L.B.); barbara.hasse@usz.ch (B.H.); 3 Division of Infectious Diseases and Hospital Hygiene, Spital Limmattal, 8952 Schlieren, Switzerland; Roberto.Buonomano@spital-limmattal.ch; 4 Division of Medicine, Spital Limmattal, 8952 Schlieren, Switzerland; alain.rudiger@spital-limmattal.ch; 5 Division of Infectious Diseases, Hospital Hygiene and Occupational Medicine, Stadtspital Triemli, 8063 Zurich, Switzerland; Gerhard.Eich@triemli.zuerich.ch; 6 Emergency Department, University Hospital Zurich, 8091 Zurich, Switzerland; Dagmar.Keller@usz.ch; 7 Division of Infectious Diseases and Hospital Epidemiology, University Children’s Hospital Zurich, 8032 Zurich, Switzerland; christoph.berger@kispi.uzh.ch

**Keywords:** SARS-CoV-2, PCR, children, saliva

## Abstract

Rising demands for repetitive SARS-CoV-2 screens and mass testing necessitate additional test strategies. Saliva may serve as an alternative to nasopharyngeal swab (NPS) as its collection is simple, non-invasive and amenable for mass- and home testing, but its rigorous validation, particularly in children, is missing. We conducted a large-scale head-to-head comparison of SARS-CoV-2 detection by RT-PCR in saliva and NPS of 1270 adults and children reporting to outpatient test centers and an emergency unit. In total, 273 individuals were tested positive for SARS-CoV-2 in either NPS or saliva. SARS-CoV-2 RT-PCR results in the two specimens showed a high agreement (overall percent agreement = 97.8%). Despite lower viral loads in the saliva of both adults and children, detection of SARS-CoV-2 in saliva fared well compared to NPS (positive percent agreement = 92.5%). Importantly, in children, SARS-CoV-2 infections were more often detected in saliva than NPS (positive predictive value = 84.8%), underlining that NPS sampling in children can be challenging. The comprehensive parallel analysis reported here establishes saliva as a generally reliable specimen for the detection of SARS-CoV-2, with particular advantages for testing children, that is readily applicable to increase and facilitate repetitive and mass testing in adults and children.

## 1. Introduction

The current gold standard for the diagnosis of Severe Acute Respiratory Syndrome Coronavirus 2 (SARS-CoV-2) infection relies on the detection by quantitative reverse-transcription polymerase chain reaction (RT-qPCR) in nasopharyngeal swabs. A range of RT-qPCRs methods have been developed and proven highly sensitive, accurate and reliable [1,2]. Nasopharyngeal swabs (NPSs) are considered the optimal material for detection, particularly in early infection [2]. However, viral load in the nasopharynx can wane in later disease stages, while the virus remains detectable in alternate specimens such as bronchioalveolar lavage or sputum, thus necessitating the validation of diagnostics tests in these specimens [3,4,5]. In addition, to overcome limitations in mass screening for early detection of SARS-CoV-2, saliva has been considered as alternate material to NPS [6,7,8,9,10]. NPS collection requires trained personnel while saliva collection is comparatively easy, needs little instruction and is amenable for self-collection. Importantly, saliva collection is non-invasive and it does not create discomfort for the patient. Saliva would, thus, be of particular advantage for testing children, who parents and pediatricians often refrain from testing due to the need to conduct a nasopharyngeal swab. Likewise, the possibility to switch to saliva would also be a relief for adults when frequent testing or large-scale screens are required. Furthermore, considering the current high level of SARS-CoV-2 testing by RT-PCR and antigen tests, which both require nasopharyngeal swabs, a shortage in swab supplies may occur. Establishing the possibility to switch to saliva collection in this situation to allow RT-PCR testing to continue is, thus, highly advisable.

Several recent studies have evaluated saliva as an alternate specimen [6,7,8,9,10,11,12,13,14,15,16,17,18,19,20,21,22,23,24,25,26,27,28,29]. While these studies generally agree that detection of SARS-CoV-2 in saliva is possible, comparative analyses came to different conclusions, with some studies noting a better performance of saliva while others found a substantially lower sensitivity. With few exceptions, patient cohorts tested thus far were, in most studies, relatively small and often included both hospitalized individuals with advanced SARS-CoV-2 infection as well as outpatients who were newly screened for infection, leaving uncertainty in which situation saliva may be best used. The overall sensitivity and, thus, utility of saliva in comparison to NPS remains, thus, differentially debated and needs to be defined. To resolve these issues, we embarked on a large-scale head-to-head comparison of saliva and NPS in a test center setting for adults and children. The high number of individuals tested (N = 1270) and the high number of positive cases detected (N = 273), paired with a true-to-life screening in test centers, empowered a highly controlled analysis which supports the applicability of saliva in routine testing and particularly provides better opportunities for testing children.

## 2. Materials and Methods

### 2.1. Study Population

Adults (N = 1100) and children (N = 170) opting for a SARS-CoV-2 test at one of five participating test centers were included. Our study included five different test sites to ensure that data are not skewed due to specific procedures at one site. Four centers were dedicated outpatient test centers (three for adults; one for children) and one was an emergency department. The study population comprised individuals with SARS-CoV-2-related symptoms based on Swiss testing criteria and asymptomatic individuals with relevant exposure to a SARS-CoV-2 index case. Hospitalized patients were not included. Individuals were included without further selection to avoid skewing. Information on symptomatic or asymptomatic status was acquired as part of the regular procedure for SARS-CoV-2 testing and reporting based on self-evaluation (asymptomatic/mild/strong) by the participants, as they did not see a physician in the test center setting.

### 2.2. Ethical Approval

The Zurich Cantonal Ethics Commission waived the necessity for a formal ethical evaluation based on the Swiss Federal Human Research Act, as the collection of saliva in parallel to a scheduled nasopharyngeal swab induces no risk (Req-2020-00398). No additional personal data beyond the usual information on symptoms and duration required by the Swiss Federal Office of Public Health (FOPH) for all SARS-CoV-2 tests in Switzerland were retrieved. Due to the ethics waiver, no informed consent had to be requested.

### 2.3. Sample Collection

Test centers were advised to use their regular swab and virus transport medium (VTM)/universal transport medium (UTM) for nasopharyngeal sampling. Transport media used by the centers included Cobas PCR Medium (Roche, Basel, Switzerland), Liquid Amies preservation medium (Copan, Bettlach, Switzerland), Virus Preservative Medium (Improviral, Singapore) and in-house VTM (HEPES (4-(2-hydroxyethyl)-1-piperazineethanesulfonic acid), Dulbecco’s Modified Eagle’s Medium (DMEM), fetal calf serum (FCS), antibiotics and antimycotics).

**Figure A1:**
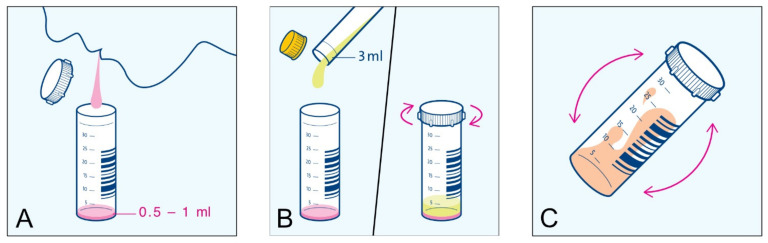
Excerpt from instruction leaflet for saliva collection. Participants were asked to clear their throat and collect approximately 0.5–1 mL saliva into a collection tube (**A**). Virus transport medium (VTM) was added to the crude saliva immediately after collection (**B**), and the content was mixed through gentle tilting (**C**).

Collection kits for saliva were supplied to the test centers: one tube for saliva collection (Sarsted 62.555.001) and a separate tube with 3 mL VTM (Axonlab AL0607). The procedure for saliva collection was described in an instruction leaflet (Appendix A, Figure A1). In Study Arm 1, “Basic”, individuals were asked to clear their throat thoroughly and collect saliva one or two times into the same tube (N = 835). As a guidance for the volume of saliva to be sampled, participants were instructed by study teams to collect 0.5–1 mL (approx. a teaspoon full). To investigate a possible influence on SARS-CoV-2 detection in saliva through differences in saliva collection, a subset of patients (N = 435) in Study Arm 2, “Enhanced”, were asked to clear their throat three times and collect saliva into the same tube. The emphasis in this study arm was on enhanced throat clearing to ascertain sampling material from the posterior oropharynx. Immediately after saliva collection, VTM was added to the crude saliva and the content was mixed through gentle tilting. Saliva was collected directly after NPS and both specimens were immediately sent for SARS-CoV-2 RT-PCR testing.

### 2.4. Quantitative SARS-CoV-2 PCR

NPS and saliva were processed identically using the procedures established for NPS in the diagnostics laboratory of the Institute of Medical Virology. Briefly, 500 uL of NPS or saliva in VTM was diluted in 500 uL of Nuclisens easyMAG Lysis Buffer (bioMérieux, Marcy l’Etoile, France), centrifuged (2000 rpm, 5 min) and analyzed with the Cobas SARS-CoV-2 IVD test (Roche) on a Cobas 6800. All testing for NPS and saliva was done in parallel on the same day. SARS-CoV-2 detection was further quantified using SARS-CoV-2 Frankfurt 1 RNA as a calibrator (European Virus Archive, 004N-02005), allowing to report both Ct (cycle threshold) and genome equivalents.

### 2.5. Verification by in-House SARS-CoV-2 E-Gene and GAPDH PCR

Discordant results of the Cobas SARS-CoV-2 test between NPS and saliva were re-analyzed using an in-house RT-qPCR targeting the E-gene based on Corman et al. [1]. GAPDH was measured as the input control as described [30]. Both assays used AgPath-ID One-Step RT-PCR chemistry (Ambion, ThermoFisher, Waltham, MA USA).

### 2.6. Data Analysis

E-gene Ct values were used for comparison. If the E-gene reported negative but ORF1 reported positive by the Cobas SARS-CoV-2 IVD test, the ORF1 result was considered and the respective sample rated positive for SARS-CoV-2. This was the case for one saliva sample. Data were analyzed using R (version 4.0.2) [31]. Furthermore, 95% confidence intervals were calculated with the epiR package (version 1.0.15). Method comparison and regression analysis (Passing–Bablok regression [32] and Bland–Altman plot [33]) were performed with the mcr package (version 1.2.1). All raw data are available in supplemental Table S1.

## 3. Results

### 3.1. Head-to-Head Comparison of Saliva and Nasopharyngeal Swabs as Material for SARS-CoV-2 Detection by RT-PCR

We sought to design a saliva collection procedure that is safe, does not create infectious waste at the collection site and does not need trained medical personnel. In order to avoid having contaminated material that needs to be disposed at the collection sites, we refrained from using collection tubes with disposable funnels and opted for collection into commonly available, cheap, wide plastic tubes. Our protocol for saliva collection instructed participants to self-collect approx. 0.5 mL saliva into an empty, wide (30 mL, 30 mm diameter) tube. Initial attempts in a pilot experiment at the participating emergency department with smaller tubes (15 mL, 17 mm diameter) showed that spitting into narrower tubes is problematic for some participants, leading to a contamination of the outside of the tube with saliva in some cases. Sampling with the wider tubes was, in contrast, unproblematic and, thus, deemed safe. Saliva sampling in children was found equally unproblematic; children were collaborating and able to expectorate. We deliberately chose to add VTM after saliva collection and not to use VTM-filled tubes as some persons feel more comfortable with sampling into empty containers. This also circumvents that the VTM is mistakenly used to gurgle or swallowed, issues that have to be considered in home testing and with children.

Saliva was mixed with VTM immediately after collection and shipped to the lab at room temperature the same day or stored at 4 °C until shipping the next day. The thus-diluted material was unproblematic for further processing in the laboratory, with no complications in pipetting, and only rarely, samples were rejected due to the viscosity of saliva or invalid results were observed.

### 3.2. High Positive Agreement of SARS-CoV-2 Detection in Saliva and Nasopharyngeal Swabs

Adults and children that qualified for a regular SARS-CoV-2 test according to the FOPH and reported to one of the participating test centers or emergency units were enrolled from 20 October 2020 to 28 January 2021. In total, 1270 individuals (male 54.6%/female 45.4%) were included (Table 1). The gender distribution in children and adults was similar. The median age was 34 with an age range of 5–98 years. Of the participants, 170 were under the age of 18. The majority of participants were symptomatic—75.6%. Days of symptoms ranged from 1 to 30, with a median of 2 days. The percentage of symptomatic individuals and participants in the Enhanced study arm was higher in children (Table 1).

**Figure 1:**
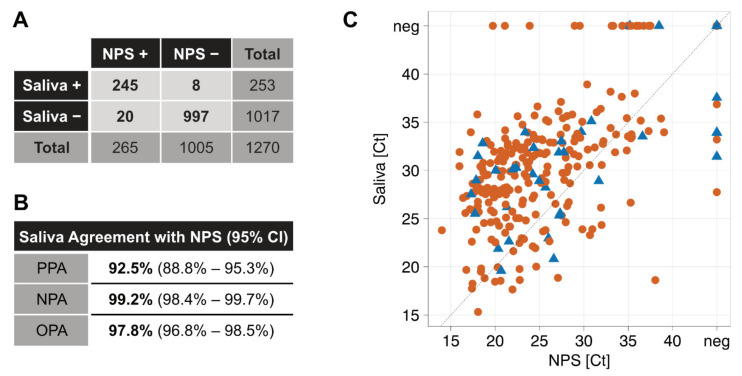
High agreement of SARS-CoV-2 detection in saliva and nasopharyngeal swabs (NPSs). (**A**) Contingency table, full cohort; (**B**) agreement values. PPA = positive percent agreement; NPA = negative percent agreement; OPA = overall percent agreement. (**C**) Summary of the full cohort (N = 1270 study participants). Roche Cobas E-gene Ct values of paired NPS and saliva samples are depicted. Red dots = adults; blue triangles = children; neg = PCR negative; black dashed line equals identity.

The SARS-CoV-2 positivity rate amongst study participants was 21.5%. Across the entire cohort and both study arms, NPS and saliva results showed a high overall percent agreement (OPA = 97.8%) and a good positive percent agreement (PPA = 92.5%, Figure 1B). In only 28 cases were discordant results observed, with 20 saliva samples and 8 NPS samples showing a negative result when the other specimen tested positive (Figure 1A). To investigate if discordant results were due to inadequate sampling, detection problems in the RT-PCR or reflective of true negatives/positives in the respective sample material, all discordant pairs were retested using an in-house RT-PCR for the E-gene in conjunction with a GAPDH measurement to control for input. Mean levels for GAPDH input were Ct = 24.3 (SD = 2.6) for NPS and Ct = 24.7 (SD = 2.1) for saliva. One false-negative saliva sample (E-gene Ct 19.7 in NPS) did not contain any material (GAPDH Ct > 40). Excluding this sample, the PPA in the NPS Ct 15–20 range reached 100% (Table 2).

Re-assessment with an in-house E-gene PCR confirmed all discordant results. For one case with a negative NPS, a second swab was collected the following day. This sample showed a high viral load, confirming an unsuccessful swab collection the day earlier.

Of note, in our head-to-head comparison, both NPS (N = 3) and saliva (N = 5; N = 4 excluding the sample that did not contain saliva) produced false-negative results in cases where the other specimen showed a high viral load (Ct < 33), highlighting variability in collection for both specimens.

Breakdown in adults and children (Appendix A, Table A1, Table A2, Table A3 and Table A4) underlines the suitability of saliva for both age groups. Of particular note, in children, SARS-CoV-2 infections were more often detected in saliva than NPS (positive predictive value = 84.8%; NPS negatives with paired positive saliva (N = 5); saliva negative with paired NPS positive pairs (N = 2)). These results suggest that difficulties in NPS sampling in children may impact the efficacy of SARS-CoV-2 detection.

### 3.3. SARS-CoV-2 Loads in Saliva and Nasopharyngeal Swab Correlate

**Figure 2:**
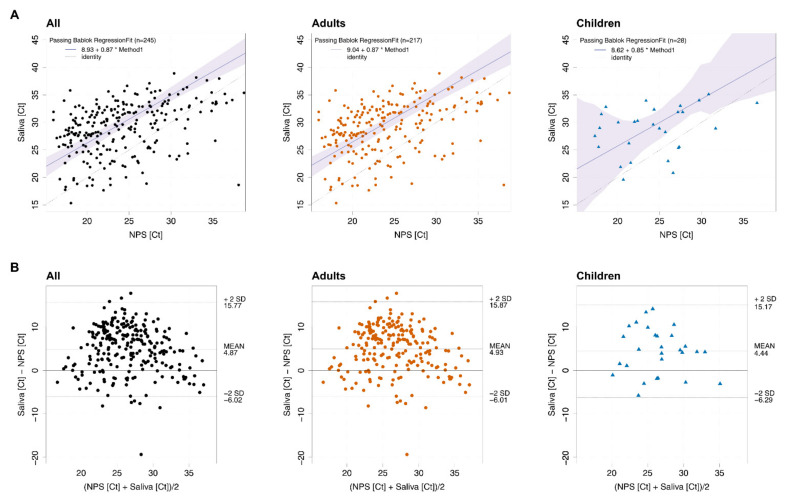
SARS-CoV-2 levels in saliva and nasopharyngeal swabs correlate. (**A**) Passing–Bablok regression of E-gene Ct values of NPS and saliva of all positive pairs from the full cohort (N = 245; *p* < 0.0001), adults (N = 217; *p* < 0.0001) and children (N = 28; *p* = 0.079). Black dashed line equals identity, blue line shows linear trend. (**B**) Bland–Altman plot of E-gene Ct values of NPS and saliva of all positive pairs from the full cohort (N = 245), adults (N = 217) and children (N = 28).

Correlation analysis of sample pairs that both tested positive (N = 245) confirmed that saliva and NPS results are in good agreement (Figure 2A). Notably, Ct values in saliva were, on average, 4.87 higher than the corresponding Ct values in NPS across the full cohort (Figure 2B, left panel). Average Ct value differences for NPS and saliva in adults and children were similar (4.93 and 4.44, respectively, Figure 2B, middle and right panel). Higher Ct values in saliva correspond to a factor of 29-, 30- and 22-times lower viral loads in saliva for the full cohort, adults and children, respectively. Of note, at high Ct values in the corresponding NPS samples, the reduction in viral load in saliva was less pronounced, possibly adding to the high rate of detection in saliva.

### 3.4. Detection of SARS-CoV-2 in Saliva from Symptomatic and Asymptomatic Individuals

**Figure 3:**
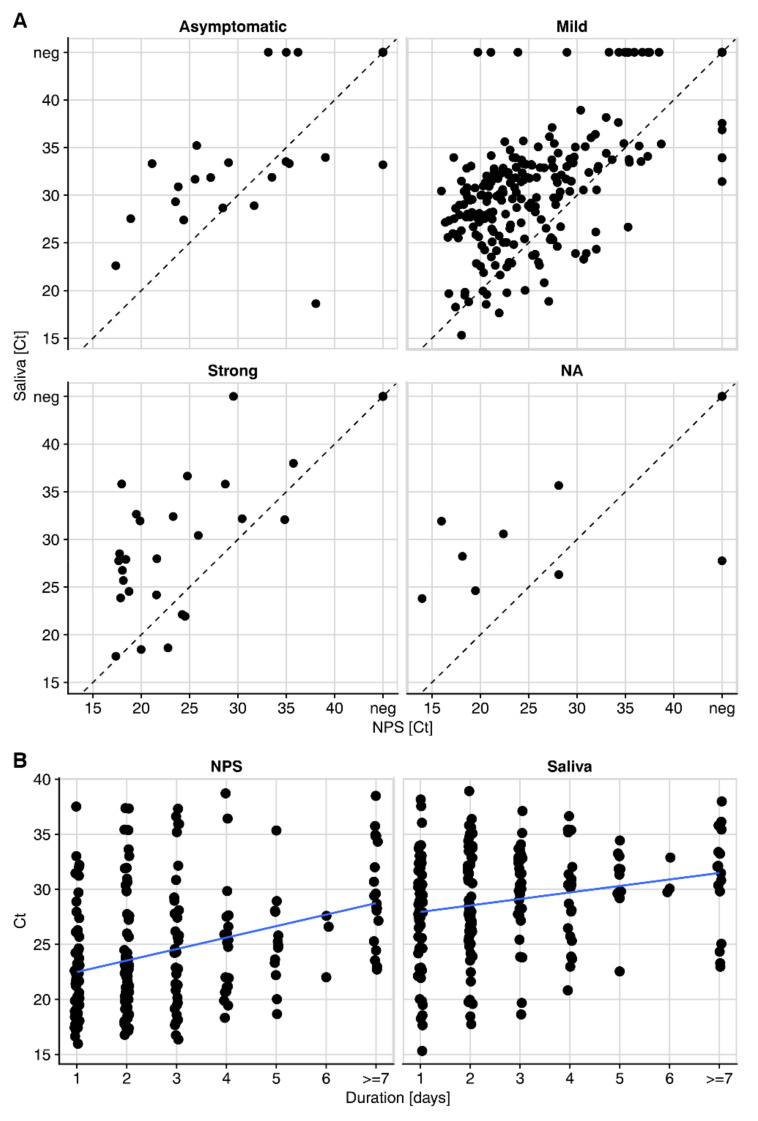
Viral loads in NPS and saliva decrease with ongoing infection. (**A**) E-gene Ct values of NPS and saliva of all pairs from the full cohort stratified by symptoms. (**B**) Duration of symptoms in symptomatic patients (N = 927) versus E-gene Ct values in saliva and NPS. neg = PCR negative; line shows linear trend.

Our study recorded the severity of symptoms (asymptomatic/mild/strong) at the sampling time point by self-evaluation (Figure 3A). We observed a good positive percent agreement of saliva and NPS in symptomatic individuals in the full cohort (PPA = 92.2%), adults (PPA = 92.9%) and children (PPA = 92.9%). In line with the trend of lower viral loads, i.e., higher Ct values in the absence of symptoms (asymptomatic median Ct, 28.7; mild symptoms median Ct, 23.5; strong symptoms median Ct, 21.6), the PPA was lower in asymptomatic participants (PPA = 85.0%). We observed decreasing viral loads with ongoing symptomatic infection in NPS, highlighting a transient window of detection in the upper respiratory tract. Interestingly, changes in saliva were, overall, less dynamic than in NPS (Figure 3B).

### 3.5. Intensified Throat Clearing with Saliva Collection Is Favorable

**Figure 4:**
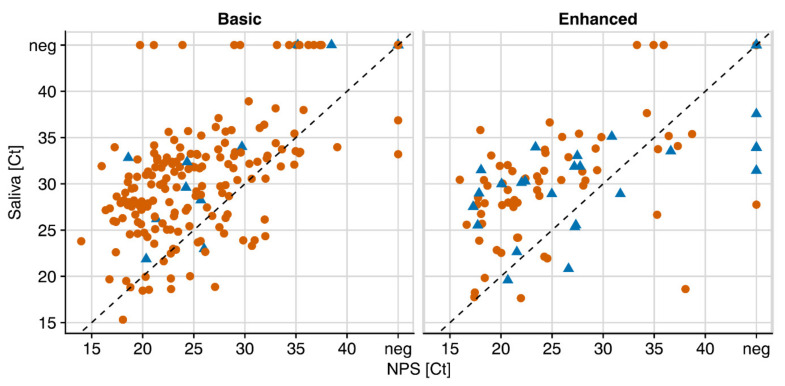
Intensified saliva sampling increases the low level of SARS-COV-2 detection in saliva. E-gene Ct values of paired NPS and saliva samples of the study arm “Basic” (1–2× saliva per tube; N = 835) and “Enhanced” saliva collection (3× saliva per tube; N = 435). Red dots = adults; blue triangles = children.

To investigate if the intensity of saliva collection has an impact, we analyzed the two study arms of saliva collection separately. Participants were either asked to clear their throat thoroughly and collect about 0.5–1 mL of saliva (“Basic”, N = 835) or, in an intensified protocol, to repeat throat clearing and spitting three times (“Enhanced”, N = 435). To ensure appropriate statistical power, we investigated the influence of sampling solely in the full cohort. We found that intensified saliva collection appears favorable for samples with low viral load. With the enhanced sampling protocol, PPA with NPS of Ct >33 reached 66.7% (CI 35–90%), compared to 50.0% (CI 28–72%) with the basic protocol (Figure 4 and Table 3). The differences were, however, not significant, highlighting robust detection of SARS-CoV-2 in saliva in the two collection procedures tested.

Based on these findings, local health authorities in Zurich, Switzerland, have launched mass testing for outbreak management utilizing the enhanced saliva sampling procedure in schools. Thus far, we have conducted mass tests in six schools (N = 350–700 tests per school), and saliva sampling proved well accepted by children, parents and teachers and easily applicable for mass collection and laboratory processing. 

## 4. Discussion

**Figure A2:**
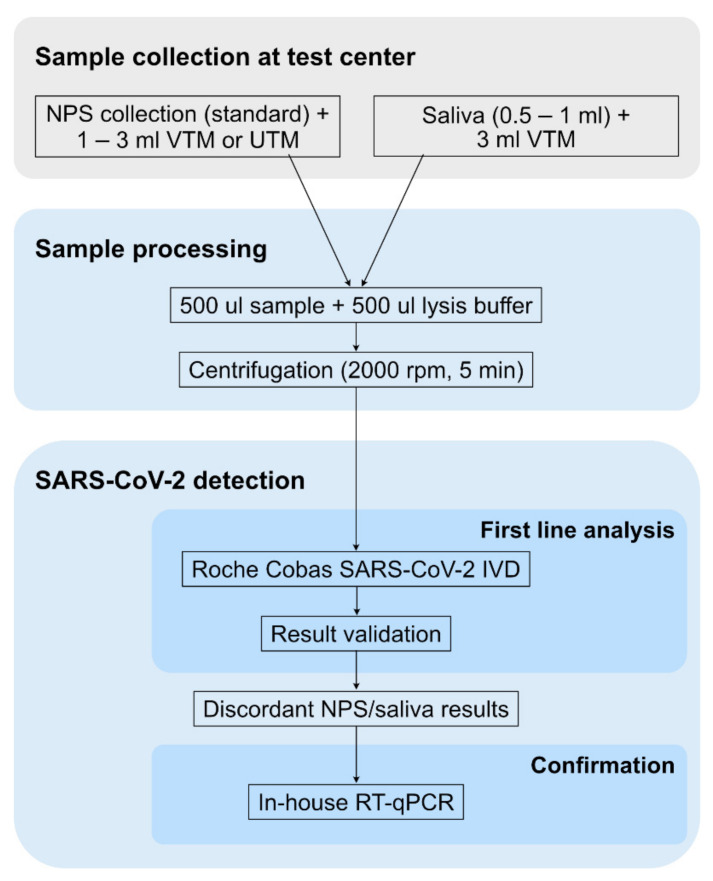
Flowchart of the sample analysis process.

In the present study, we sought to devise and evaluate a saliva sampling strategy that provides (i) representative sampling of virus-containing material, (ii) easy and safe collection, (iii) comfort for repetitive testing in adults and children, (iv) possibility for home collection and (v) straightforward processing in the laboratory (a flowchart of the procedure is included Appendix B, Figure A2). As a key element, saliva collection should not create infectious waste and should not need supervision by medical personnel to make it amenable for safe collection at home or in public institutions—for instance, schools. We opted for a saliva collection procedure where participants clear their throat to first generate saliva from the back of the throat and then expectorate the saliva into an empty container. We considered clearing the throat important to sample material from the posterior oropharynx, where SARS-CoV-2 sampling by oropharyngeal swabs is known to be efficient [34,35]. While gargling with saline or buffer solutions has been suggested as a possibility to sample saliva from the deep throat [36,37], we rated this procedure as less operable as the gargling solution would need to be optimized for taste to be accepted by individuals, could not include preservatives and gargling itself may potentially generate aerosols. In addition, gargling is not practicable for many younger children, for whom we particularly sought to create more possibilities for SARS-CoV-2 testing, as NPS collection is often not practical in children. 

Our study demonstrates an excellent agreement of saliva in the head-to-head comparison with NPS and, thus, recommends saliva as alternate material for SARS-CoV-2 detection by RT-PCR. Up to Ct 33 (equivalent to approximately 26,000 genome copies/mL) in the corresponding NPS, a notably high PPA (97.8% for the full cohort), is reached. Of note, virus loads in an even lower range are considered to impose a marginal risk for transmission as suggested by contact tracing and in vitro culturing studies [38,39,40]. A decrease in sensitivity as observed for saliva testing by RT-PCR in the current study is, thus, acceptable, and the advantages outweigh the drawbacks from low-level positives not detected. Saliva performed equally well in children (PPA = 93.3%); notably, more saliva samples tested positive compared to NPS in this population (positive predictive value, PPV = 84.8%). The reduced efficacy of NPS in children underlines the difficulty in obtaining correct swabs from them, highlighting the potential of saliva collection particularly when diagnosing children. The overall performance of saliva was remarkable considering that we and others observed lower viral loads in saliva compared to NPS [22,41]. 

In view of the observed PPA in detection, saliva may safely be envisaged as a substitute for NPS detection in a range of settings. Possible scenarios include (i) sampling of children, (ii) home collection, (iii) test centers without trained medical personnel (e.g., schools, universities and companies), (iv) a non-irritating alternative for persons that need frequent testing due to their occupation or health status and (v) repetitive mass testing. In situations where other respiratory viruses besides SARS-CoV-2, e.g., Influenza and Respiratory syncytial virus (RSV), need to be excluded, NPS should, however, remain the standard material of choice as it allows rapid detection with multiplex-PCR from a single specimen. In addition, if SARS-CoV-2 infection has to be ruled out with the highest possible sensitivity (e.g., in transplantation), NPS should equally continue to be used.

The majority of SARS-CoV-2 in saliva represents likely virus secreted from infected cells in the nasopharynx and is not locally produced. Collecting material from the posterior oropharynx may, thus, be important. This is also highlighted in our study as the collection protocol with intensified throat clearing showed a trend to increase PPA at low viral loads.

It remains possible that eating or drinking shortly before collection may decrease viral content in the oral cavity and throat. In the present study, neither eating, drinking nor smoking were controlled as study subjects came for an elective analysis by NPS and, thus, could only be informed about the saliva sampling on site immediately before the collection. Abstaining from food and beverage uptake shortly (1 h) before saliva collection could be considered in forthcoming applications of saliva as a test material, as it may increase the efficacy of SARS-CoV-2 detection in saliva even further.

In summary, our analysis rates saliva as a valid alternate specimen for SARS-CoV-2 detection by RT-PCR, which is even superior in children when compared to NPS. Saliva collection is non-invasive, not strenuous for patients, does not need trained personnel, allows collection at any location and allows self-collection. Importantly, as we show here, saliva collection does not require any adjustments in the diagnostics tests; established RT-qPCR can be used. Of note, while using RT-PCR as a method for saliva testing causes only a minor loss in sensitivity compared to NPS, loss in sensitivity for other test systems must be carefully weighed and analyzed, as methods with generally lower sensitivity than RT-PCR, such as antigen tests and isothermal PCR methods, may depend on testing of NPS to sustain a high enough sensitivity. Future developments that allow sensitive detection of SARS-CoV-2 in saliva by rapid antigen tests and isothermal PCR would, however, have immense potential, as the ease in sample collection and rapid and inexpensive testing would provide important advantages for mass testing [42,43,44,45]. Saliva RT-PCR testing has a further advantage over screens based on rapid antigen tests, as RT-PCR collected material allows for immediate subsequent analysis of positives as currently necessary for variant tracking by PCR and surveillance sequencing. Combined with the high reliability in detecting SARS-CoV-2 infection as demonstrated in our head-to-head comparison with the standard NPS, increasing and facilitating test efforts by monitoring SARS-CoV-2 infection in saliva is rapidly attainable. Saliva combined with RT-PCR, particularly, should be considered to improve testing opportunities in children as a means to circumvent school closing.

## Figures and Tables

**Table 1:** Participant demographics.

	Total (*n* = 1270)	Adults (*n* = 1100)	Children (*n* = 170)
Male/Female (%)	693 (54.6%)/577 (45.4%)	605 (55%)/495 (45%)	88 (51.8%)/82 (48.2%)
Age median (range)	34 (5–98)	37 (18–98)	13 (5–17)
Symptomatic mild (%)	836 (65.8%)	701 (63.7%)	135 (79.4%)
Symptomatic strong (%)	91 (7.2%)	79 (7.2%)	12 (7.1%)
Asymptomatic (%)	299 (23.5%)	279 (25.4%)	20 (11.8%)
No information on symptoms (%)	44 (3.5%)	41 (3.7%)	3 (1.76%)
Median days of symptoms (range)	2 (1–30)	2 (1–30)	2 (1–21)
“Basic”/“Enhanced” study arm	835 (65.7%)/435 (34.3%)	783 (71.2%)/317 (28.8%)	52 (30.6%)/118 (69.4%)

**Table 2:** Positive percent agreement (PPA) stratified by NPS E-gene Ct values.

NPS (Ct)	>10–15	>15–20	>20–25	>25–30	>30–33	>33–35	>35–40
NPS positive	1	59	96	60	15	13	21
Saliva false negative	0	1 (0 *)	2	2	0	5	10
PPA	100%	98.3% (100% *)	97.9%	96.7%	100%	61.5%	52.4%

* Excluding one sample that did not contain saliva as defined by GAPDH measurement.

**Table 3:** Positive percent agreement (PPA) stratified by NPS E-gene Ct values and saliva sampling.

	Full Cohort (N = 1187)			Basic Sampling (N = 835)			Enhanced Sampling (N = 352)		
NPS (Ct)	all	>10–33	>33–40	all	>10–33	>33–40	all	>10–33	>33–40
NPS positive	265	231	34	183	161	22	82	70	12
Saliva false negative	20	5	15	16	5	11	4	0	4
PPA	92.5%	97.8%	55.9%	91.3%	96.9%	50.0%	95.1%	100%	66.7%

## Data Availability

All raw data are provided as supplementary materials.

## Supplementary Materials

The following are available online at https://www.mdpi.com/2076-2607/9/3/642/s1, Table S1: Raw data.

## Appendix A

**Table A1:** Contingency Table: Adults.

	NPS +	NPS −	Total
Saliva +	217	3	220
Saliva −	18	862	880
Total	235	865	1100

**Table A2:** Contingency Table: Children.

	NPS +	NPS −	Total
Saliva +	28	5	33
Saliva −	2	135	137
Total	30	140	170

**Table A3:** Agreement values: Adults.

Saliva Agreement with NPS (95% CI)	
PPA	92.3% (88.2–95.4%)
NPA	99.7% (99.0–99.9%)
OPA	98.1% (97.1–98.8%)

PPA = Positive Percent Agreement; NPA = Negative Percent Agreement; OPA = Overall Percent Agreement.

**Table A4:** Agreement values: Children.

Saliva Agreement with NPS (95% CI)	
PPA	93.3% (78.0–99.2%)
NPA	96.4% (91.9–98.8%)
OPA	95.9% (91.7–98.3%)

PPA = Positive Percent Agreement; NPA = Negative Percent Agreement; OPA = Overall Percent Agreement.
